# Methyltransferase-like 3-mediated RNA N^6^-methyladenosine contributes to immune dysregulation: diagnostic biomarker and therapeutic target

**DOI:** 10.3389/fimmu.2025.1523503

**Published:** 2025-03-24

**Authors:** Deshuang Zhang, Ting Xu, Xiaoxue Gao, Yi Qu, Xiaojuan Su

**Affiliations:** ^1^ Key Laboratory of Birth Defects and Related Diseases of Women and Children (Ministry of Education), West China Second University Hospital, Sichuan University, Chengdu, Sichuan, China; ^2^ Division of Neonatology, Department of Pediatrics, The Affiliated Hospital of Southwest Medical University, Luzhou, Sichuan, China; ^3^ Department of Pediatrics, School of Clinical Medicine & The First Affiliated Hospital of Chengdu Medical College, Chengdu, China

**Keywords:** methyltransferase-like 3, immune dysfunction, antiviral immunity, immune tolerance, diagnostic biomarker, therapeutic target

## Abstract

Methyltransferase-like 3 (METTL3) plays a crucial role in post-transcriptional gene regulation. Substantial evidence links METTL3 to various immune dysfunctions, such as the suppression of antiviral immunity during viral infections and the disruption of immune tolerance in conditions like autoimmune diseases, myeloid leukemia, skin cancers, and anticancer immunotherapy. However, a thorough review and analysis of this evidence is currently missing, which limits the understanding of METTL3’s mechanisms and significance in immune dysfunctions. This review aims to elucidate the roles and mechanisms of METTL3 in these immune issues, highlighting its connections and proposing new insights into its modulation of immune responses. Analysis results in this review suggest that METTL3 hampers antiviral immunity, worsens viral replication and infection, and disrupts immune tolerance; conversely, regulating METTL3 enhances antiviral immunity and facilitates viral clearance. Moreover, clinical data corroborates these findings, showing that METTL3 overexpression is associated with increased susceptibility to viral infections and autoimmune conditions. This review establishes a theoretical basis for considering METTL3 as a novel regulator, an important diagnostic biomarker, and a potential target for treating immune dysfunctions.

## 1 Introduction

The immune system primarily differentiates between healthy and atypical cells to mitigate the risk of infections (1). However, dysfunctions can occur due to a variety of factors, including genetic immunopathology, immunosuppressive infections such as human immunodeficiency virus (HIV), hypersensitivity reactions, and autoimmune disorders (2–4). Consequently, a thorough elucidation of the complex mechanisms that underlie immune system impairments is essential for addressing a range of immunological issues, from infectious diseases to malignancies.

**Figure 1:**
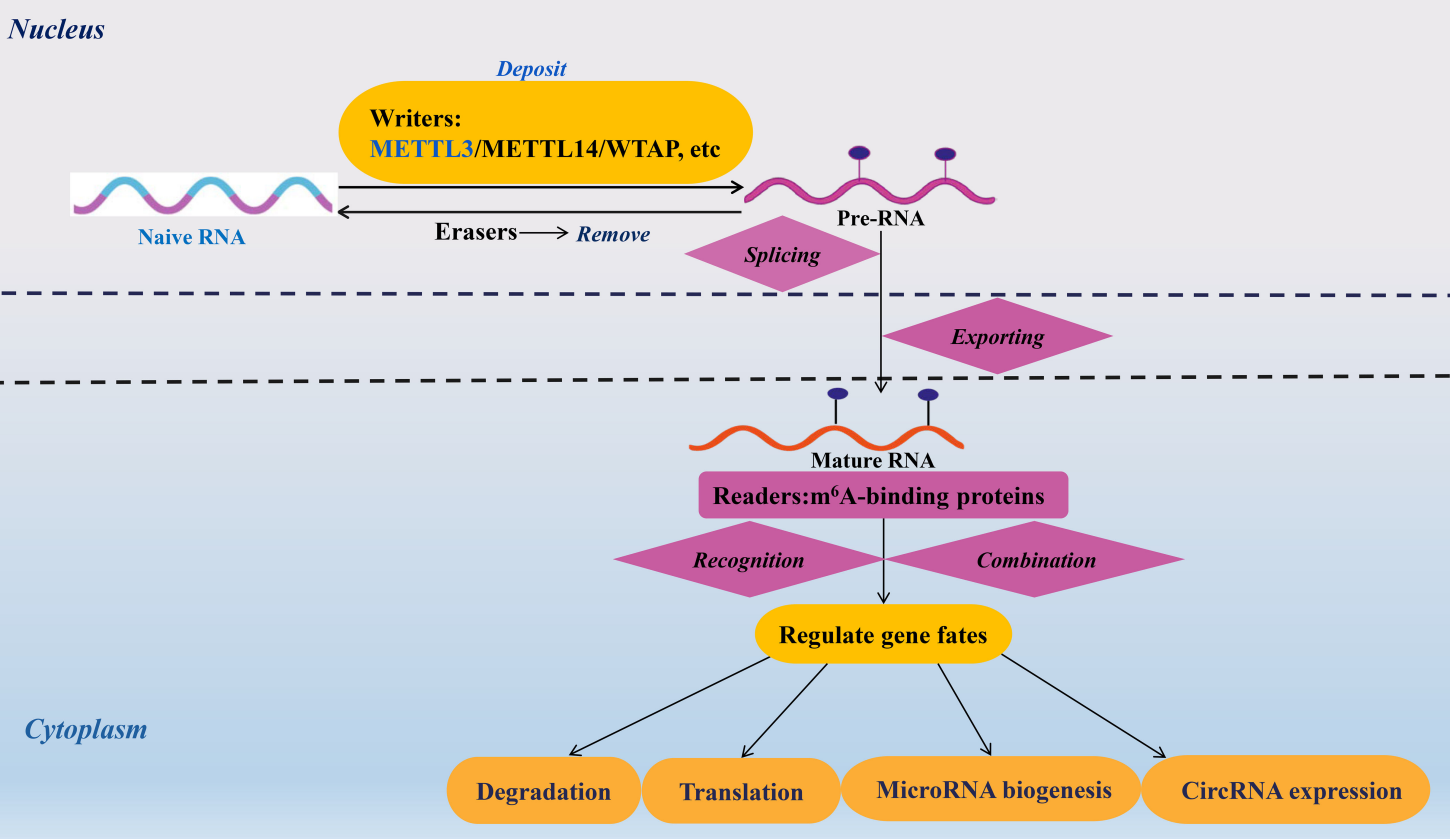
Mechanisms and roles of m^6^A modification on RNAs. The m^6^A modification of RNA modulates the fate of target transcripts through the action of m^6^A methyltransferases, demethylases, and binding proteins. The roles of m^6^A modification in RNA encompass processes such as splicing, nuclear transport, degradation, translation, microRNA maturation, and the expression of circular RNAs. Methyltransferase-like 3/14(METTL3/14). Wilms tumor 1-associated protein (WTAP). N^6^-methyladenosine (m^6^A).

In recent years, RNA N^6^-methyladenosine (m^6^A), a dynamic and reversible epigenetic modification, has emerged as a pivotal regulator of immune-related diseases, including viral infections, inflammatory disorders, cancers, and autoimmune dysregulation (5–9). This post-transcriptional mechanism is orchestrated by three classes of proteins: “writers” that install m^6^A marks, “erasers” that remove them, and “readers” that interpret these modifications to direct RNA fate (10–12). The writer complex, composed of METTL3 (the catalytic core), METTL14, and WTAP, deposits m^6^A onto RNA, while erasers such as FTO and ALKBH5 erase these marks (13–15). Readers, including YTHDF and IGF2BP proteins, then bind m^6^A sites to regulate RNA splicing, stability, translation, and other functional outcomes (16, 17). Together, these players fine-tune gene expression, linking m^6^A to diverse immunological processes (**Figure 1**).

Central to this regulatory system is METTL3, the principal methyltransferase driving m^6^A deposition (18, 19). An increasing number of investigations have elucidated the involvement of METTL3 in immune dysregulation, encompassing the attenuation of antiviral immunity (in the context of viral infections) and the perturbation of immune tolerance (observed in autoimmune disorders, myeloid leukemia, cutaneous malignancies, and anticancer immunotherapy). Furthermore, the intricate role of METTL3 in modulating immune responses extends beyond mere dysregulation; it also highlights the potential for therapeutic intervention. Recent studies have shown that METTL3 not only influences the expression of immune-related genes but also interacts with various signaling pathways that govern immune cell function. For instance, METTL3 has been implicated in the regulation of the NF-κB signaling pathway, which is crucial for the activation of immune responses (20). By modifying the mRNA methylation landscape, METTL3 can alter the stability and translation of mRNAs encoding key proteins involved in inflammation and immune activation, thus shaping the overall immune response (20). In addition to its role in antiviral immunity, METTL3 has been linked to the maintenance of immune tolerance, particularly in the context of autoimmune diseases. For example, its dysregulation can lead to the aberrant activation of T cells, which is a hallmark of autoimmune pathology (21). Recent literature indicates that targeting METTL3 could restore normal immune tolerance mechanisms, offering a novel approach to treat autoimmune conditions. The potential of METTL3 as a therapeutic target is further underscored by investigations exploring the effects of METTL3’s inhibitors, which have demonstrated promising results in preclinical models of autoimmune diseases (22, 23). Moreover, the relationship between METTL3 and immune-related malignancies such as myeloid leukemia and cutaneous malignancies reveals the dual role of METTL3 in both tumor promotion and immune evasion (22, 23). Studies have shown that METTL3 can enhance the expression of oncogenes while simultaneously repressing tumor suppressor genes through mRNA methylation (22, 23). This duality underscores the complexity of METTL3’s function in the tumor microenvironment, where it not only influences cancer cell survival and proliferation but also modulates the immune landscape, potentially leading to immune escape mechanisms. In the context of anticancer immunotherapy, the modulation of METTL3 activity may provide new avenues for enhancing therapeutic efficacy. For instance, by restoring proper METTL3 function, it may be possible to reinvigorate exhausted T cells and enhance their ability to target tumor cells effectively. Recent clinical trials are beginning to explore the combination of METTL3 modulators with existing immunotherapeutic agents, aiming to synergistically improve patient outcomes.

Despite the extensive exploration of METTL3’s functions in immune dysfunctions, a thorough review that examines their interconnections and offers insights into the prospective applications (diagnostic, therapeutic, and prognostic) of METTL3 remains absent. This review aims to synthesize findings that underscore the roles and mechanisms of METTL3 in modulating immune-related pathological processes to elucidate their interrelations. Additionally, we evaluate the potential of METTL3 as a viable target for immunological diagnostics, therapeutics, and prognostic assessments.

## 2 Association between METTL3 and antiviral immunity

### 2.1 METTL3 and antiviral immunity

Infections reflect the balance between intracellular virulence factors and host defense mechanisms (24). Virulence factors infection and host defense involve the innate and adaptive immune mechanisms, which are anti-immune responses to inflammation (25). Previous studies have demonstrated that viral RNA contains internal METTL3-mediated m^6^A modifications. For example, Xu et al. (26) reported that DEAD-box helicase 5 (DDX5) interacts with METTL3 following viral infection to promote the decay of IκB kinase γ and p65 mRNAs in an YTH N^6^-methyladenosine RNA binding protein 2 (YTHDF2)-dependent manner while promoting DExD/H-box helicase 58 (DHX58) translation and inhibiting the antiviral innate response. Therefore, DDX5 and METTL3 play pivotal roles in reducing antiviral innate immunity through their ability to block the p65 pathway while activating the DHX58/TANK-binding kinase 1 pathway. Chen et al. (27) found that the upregulated expression of METTL3 increases porcine epidemic diarrhea virus (PEDV) replication, which depends on YTHDF1 modulating the stability of m^6^A-modified PEDV RNA (27). However, regarding host innate immunity, Zhang et al. (28) suggested that YTHDF3, rather than METTL3, collaborates with poly (A)-binding protein 1(PABP1) and eukaryotic translation initiation factor 4 gamma 2 (eIF4G2) to facilitate forkhead box O3 (FOXO3) translation by binding to the translation initiation region of FOXO3 mRNA, thereby impeding antiviral immunity under homeostatic conditions (**Table 1**).

**Table 1:** Targets and signaling pathways of METTL3’s function in modulating antiviral immune responses and facilitating pathogen invasion.

METTL3			
Targets	Signaling pathways	Outcomes	Reference
*IκB kinase γ and p65, DHX58*	p65 pathway; DHX58/TANK-binding kinase 1 pathway	Reducing antiviral innate immunity	(26)
*PEDV*	METTL3/YTHDF1-PEDV axis	PEDV replication and infection	(27)
*FOXO3*	METTL3/PABP1/eIF4G2-FOXO3 axis	Impeding antiviral immunity	(28)
*EV71*	Subcellular localization of METTL3	EV71 replication and infection	(29)
*RdRp*	METTL3-RdRp-EV71 axis	EV71 replication and infection	(30)
*IL-1beta, TNF-a, IL-6, IL-18, COX-2 p65*	Proinflammatory	Inflammatory bowel disease	(31)
*TEAD1*	METTL3/YTHDF1-TEAD1 axis	Inflammatory bowel diseases	(32)
*HIV-1*	METTL3/YTHDC1-HIV-1 axis	HIV-1 infection	(33)
*EBNA2, BHRF1*	METTL3-EBNA2/BHRF1 axis	Ebola virus infection	(34)

METTL3 attenuates antiviral immune responses, thereby facilitating viral replication and dissemination through various mechanisms.

Methyltransferase-like 3 (METTL3). Porcine epidemic diarrhea virus (PEDV). YTH N^6^-methyladenosine RNA binding protein 1 (YTHDF1). Poly (A)-binding protein 1(PABP1). Eukaryotic translation initiation factor 4 gamma 2 (eIF4G2). Forkhead box O3 (FOXO3). Enterovirus type 71 (EV71). RNA-dependent RNA polymerase (RdRp). Interleukin (IL). Tumor necrosis factor-α (TNF-a). Transcriptional enhanced associate domain 1 (TEAD1). YTH N^6^-methyladenosine RNA binding protein 1 (YTHDF1). Human immunodeficiency virus (HIV). YTH domain containing 1 (YTHDC1). Epstein-Barr nuclear antigen 2 (EBNA2). BamHI-H rightward open reading frame 1 (BHRF1).

Another study evaluates the prevalence of m^6^A modification in the enterovirus type 71 (EV71) genome within human cells and identifies a conserved, preferred site for modification across various viral strains (29). That is, a single m^6^A modification at the junction between 5’-untranslated regions and rotavirus (RV) subunit VP4 is found to have translational functionality. During EV71 infection, the level of METTL3 expression is upregulated and is specifically localized in the cytoplasm to intermediate viral double-stranded RNA (dsRNA) replication (29). The upregulated expression of METTL3 results in a significant induction of EV71 replication, whereas depletion of the METTL3 results in a significant reduction of EV71 replication. It is noteworthy that both EV71 and METTL3 utilize common nuclear import proteins. The nuclear translocation of METTL3 is facilitated by greater binding to its nuclear localization sequence. The findings of this investigation unveil an intrinsic mechanism through which EV71 modulates the subcellular localization of METTL3 to enhance its gene expression, thereby enhancing our comprehension of RNA epi-transcriptomic during the EV71 replication cycle. Additionally, Hao et al. (30) revealed that METTL3 leads to enhanced sumoylation and ubiquitination of the viral RNA-dependent RNA polymerase (RdRp) that subsequently promotes EV71 replication. Taken together, these findings demonstrate that the host m^6^A modification complex interacts with viral proteins to modulate EV71 replication (**Table 1**).

Intestinal stem cells (IECs) are multipotent adult stem cells that continuously differentiate into specialized cells of the intestinal epithelium to maintain intestinal functions (35). Dysregulated cytokine secretion and signal transduction mechanisms via IECs contribute to inflammatory bowel disease (IBD) (35). Yang et al. (31) reported that the expression of METTL3 is significantly upregulated in IBD. METTL3 upregulation enhances cell viability, suppresses cell apoptosis, reduces cleavage of apoptotic caspase 3 and caspase 9, and increases the production of proinflammatory cytokines (interleukin [IL]-1beta, tumor necrosis factor-α [TNF]-a, IL-6, and IL-18) and inflammatory enzymes (COX-2 and inducible nitric oxide synthase), as well as of p65 phosphorylation (31). Furthermore, Jiang et al. (32) found that METTL3 and YTHDF1 inhibit transcriptional-enhanced associate domain 1 (TEAD1) translation in IECs, thereby hindering its differentiation. These findings demonstrate that the METTL3/YTHDF1-TEAD1 axis triggers IEC-induced IBD. Therefore, the abnormally enhanced expression of METTL3 exacerbates IBD (**Table 1**).

The function of the prototypical immunodeficiency virus, HIV-1, specifically targets T cells and their elimination facilitates secondary infections by other pathogens, is also intricately linked to METTL3-mediated RNA m^6^A modification (36). A study demonstrated that the activation of the METTL3/METTL14/WTAP RNA methyltransferase complex promotes viral particle production in cells harboring HIV-1 provirus (37). Furthermore, both the m^6^A methylation level of HIV-1 RNA and the number of m^6^A residues in the host cell are elevated. Another study revealed that the METTL3/METTL14 methyltransferase complex and cytoplasmic YTHDF2 work in tandem to suppress the abundance of HIV-1 RNAs in infected cells (33). YTH domain containing 1 (YTHDC1) knockdown in HIV-1-producing cells enhances the production of unsliced and incompletely spliced HIV-1 RNAs while leaving the levels of multiply spliced transcripts and the nuclear-cytoplasmic distribution of viral transcripts unaffected (33). YTHDC1 specifically binds to HIV-1 transcripts in a METTL3-dependent manner. Together, these findings suggest that METTL3, YTHDF2, and YTHDC1 are negatively correlated with HIV-1. In addition to their impact on HIV-1, METTL3-mediated RNA m^6^A modification affects Ebola virus infection. For instance, Zheng et al. (34) demonstrated that following Ebola virus infection, host cellular METTL3-mediated RNA m^6^A modification significantly enhances the expression of the viral proteins, epstein-barr nuclear antigen 2(EBNA2), and BamHI-H rightward open reading frame 1(BHRF1) (**Table 1**).

### 2.2 METTL3 suppression and antiviral immunity

The high level of METTL3 triggers virus replication and inflammatory cascade. Therefore, targeted downregulation of METTL3 might improve the capacity of antiviral immunity and virus clearance (38). Specifically, RV is a nonenveloped icosahedral-structured virus with 11 segments of dsRNA (39). RV encodes multiple viral proteins to inhibit innate immune responses by degrading interferon regulatory factors (IRFs) and mitochondrial antiviral-signaling proteins, thus facilitating efficient virus infection and replication (40). Therefore, the timely induction of an IFN response is key to the host’s successful control of invading viruses. METTL3-mediated RNA m^6^A modification plays a role in these processes (**Table 2**).

**Table 2:** Downregulation of METTL3 improves antiviral immunity and promotes viral clearance.

METTL3			
Targets	Signaling pathways	Outcomes	Reference
*IRF7*	METTL3-IRF7-interferon I/III antiviral signaling cascade	Restricts RV infection	(40)
*IFN-beta*	METTL3-IFN-beta-type I interferon axis	Inactivated virus infection	(41)
*Nuclear METTL3*	Nuclear translocation of METTL3	Impedes SARS-CoV-2 and HCoV-OC43 replication	(42)
*Inflammatory gene RIG-I*	Innate immune signaling pathways	Severe coronavirus disease	(43)
*Virus-derived transcripts*	Antiviral immune signaling	Evade host innate immunity	(44)
*Type I interferon*	RIG-I-METTL3-Type I interferon axis	Expedites the clearance of the RNA virus, vesicular stomatitis virus	(45)
*RIG-like receptors*	METTL3-RIG-I/MDAP5 axis		
*Suppress Viral dsRNA formation*	Innate sensing pathways of dsRNA		

Targets, mechanisms, and outcomes of METTL3 downregulation in antiviral immunity are comprehensively summarized and analyzed. Low levels of METTL3 improve antiviral innate immunity and promote viral clearance in adaptive immunity via different targets and mechanisms.

Methyltransferase-like 3 (METTL3). Interferon regulatory factors (IRFs). Interferon-beta (IFN-beta). Retinoic acid-inducible gene I (RIG-I). Melanoma differentiation-associated protein 5 (MDAP5). Double-stranded RNAs (dsRNAs).

METTL3 expression is suppressed in response to viral infection or cell stimulation with an inactivated virus, leading to the activation of interferon-beta (IFN-beta), which encodes the primary cytokine responsible for driving type I IFN responses by enhancing its stability (41). Wang et al. (40) discovered that targeted deletion of METTL3 in the IECs not only protects against RV infection but also increases the levels of IFNs and IFN-stimulated genes. Mechanically, METTL3 depletion promotes IRF7 mRNA stability and increases the expression of type I/III interferon. These findings reveal a novel METTL3-IRF7-interferon I/III antiviral signaling cascade that restricts RV infection *in vivo* (**Table 2**).

Depletion of METTL3 impedes the replication of severe acute respiratory syndrome coronavirus 2 (SARS-CoV-2) and the beta coronavirus, human coronavirus -OC43 (HCoV-OC43) (42). Furthermore, infection with HCoV-OC43 facilitates the nuclear translocation of METTL3, thereby indicating the potential of targeting the METTL3-mediated m^6^A modification as a therapeutic approach to impede coronavirus replication (42). Li et al. (43) discovered that in patients with severe coronavirus disease caused by SARS-CoV-2, METTL3 expression is diminished, whereas inflammatory genes are activated. This occurred through the increased retinoic acid-inducible gene I (RIG-I) binding and subsequent enhancement of downstream innate immune signaling pathways and inflammatory gene expression. Furthermore, METTL3 knockdown in representative members of the *Pneumoviridae*, *Paramyxoviridae*, and *Rhabdoviridae* families significantly increases the levels of type I IFN in a RIG-I-dependent manner (44). These findings collectively suggest that RNA viruses employ METTL3-mediated m^6^A modification as a common strategy to evade host innate immunity. Thereby rendering METTL3 as a potential intervention target for improving antiviral immunity capacity (**Table 2**).

METTL3 knockdown in monocytes or hepatocytes facilitates the expression of type I IFN and expedites the clearance of the RNA virus, vesicular stomatitis virus (46). Regarding the METTL3-mediated antiviral innate immune response, Qiu et al. (45) demonstrated that METTL3 translocates into the cytoplasm upon infection by vesicular stomatitis virus to enhance m^6^A modification on viral transcripts and suppresses viral dsRNA formation, thereby attenuating the virus-sensing efficacy of RIG-like receptors, such as RIG-I and melanoma differentiation-associated protein 5 (MDAP5), while reducing antiviral immune signaling. These findings suggest that METTL3-mediated m^6^A modification on viral RNAs negatively regulates innate sensing pathways of dsRNA and that METTL3 is a potential therapeutic target for modulating antiviral immunity (**Table 2**).

## 3 Association between METTL3 and immune tolerance disruption

The immune system is generally tolerant to self-antigens and does not typically attack the cells, tissues, or organs of the body (47). However, the loss of this tolerance, often due to immune deficiencies, leads to disorders such as autoimmune diseases or food allergies (48). Immune tolerance disruption refers to the lack of an immune response against a specific antigen, which occurs when adaptive immune cells that recognize host cells persist unchecked (49). This process is amplified by the recruitment and activation of other immune cells (50). This loss of immune tolerance is detrimental to human health and is linked to METTL3-mediated RNA m^6^A modification (51).

### 3.1 METTL3 and autoimmune diseases

Autoimmune diseases arise from the breakage of self-tolerance, including autoimmune thyroid disease (AITD) (52), Graves’ disease (53), systemic lupus erythematosus (SLE) (54), rheumatoid arthritis (RA) (55), primary Sjögren’s syndrome (pSS) (56), autoimmune uveitis (57), and myasthenia gravis (MG) (58), which have a strong genetic basis (59). With advances in gene sequencing, researchers have gained a better understanding of the underlying factors that contribute to autoimmune diseases (60).

#### 3.1.1 METTL3 and AITD

Song et al. (61) revealed a significant association between hypothyroidism and the distribution of METTL3 polymorphisms at locus rs3752411 in patients with Hashimoto’s thyroiditis. These findings provide evidence for the correlation between METTL3 mutations and predisposition to AITD and suggest the potential of METTL3 as a therapeutic target for AITD treatment. Another study revealed that both METTL3 and suppressor of cytokine signaling (SOCS) are abnormally expressed in thyroid tissues and CD4+ T cells in Graves’ disease (62). METTL3 knockdown results in the upregulation of SOCS family members (SOCS1, SOCS2, SOCS4, SOCS5, and SOCS6) (62). This study also provides further evidence for the correlation between METTL3-mediated m^6^A modification and AITD (**Table 3**).

**Table 3:** Targets and functions of METTL3 in autoimmune diseases.

METTL3			
Targets	Functions	Disease	Reference
*METTL3*	METTL3 polymorphisms at locus rs3752411	**AITD**	(61)
*SOCS1, SOCS2,SOCS4, SOCS5, SOCS6 mRNA*	m^6^A modification on SOCS family members		(62)
*Foxp3 mRNA*	Enhances *Foxp3 mRNA* decay	**SLE**	(63)
*NPR3, GHR mRNA*	m^6^A modification on *NPR3* and *GHR mRNA* in an IGFBP2 dependent manner	**RA**	(64)
*IL-6, MMP-3, and MMP-9*	Activates the NF-kB signaling pathway		(65)
*TTC4 mRNA*	Enhances *TTC4 mRNA* stability and expression		(66)
*ICAM2 mRNA*	m^6^A methylation on *ICAM2 mRNA*		(67)
*METTL3 mRNA*	Associated with C3	**pSS**	(68)
*ASH1L mRNA*	Enhances *ASH1L mRNA* stability in an YTHDC2 dependent manner	**Autoimmune uveitis**	(69)
*MiR-338-3p*	Mediates miR-338-3p upregulation in activated DCs		(70)
*NLRP3 mRNA*	Activates NLRP3 inflammasome	**MG**	(71)
*PTX3 mRNA*	Decreases *PTX3 mRNA* stability		(72)

METTL3 contributes to the pathogenesis of autoimmune diseases by regulating targets differently.

The bold words indicates the specific autoimmune diseases.

#### 3.1.2 METTL3 and SLE

SLE is a chronic autoimmune disease, which might be triggered by the dysfunction of immune cells such as T cells, B cells, monocytes, neutrophils, and dendritic cells (73). The current perspective from the m^6^A modification for the pathogenesis and treatment failure of SLE has enhanced the mechanism understanding of SLE. For example, Luo et al. (74) demonstrated that the METTL3 mRNA levels in peripheral blood from patients with SLE are significantly decreased, which suggests a correlation between METTL3 and patients with SLE. Additionally, excessive activation of CD4+T lymphocytes and imbalanced effector T-cell differentiation contribute to SLE pathophysiology (74). Lu et al. (63) found that METTL3 is at a low level in CD4+ T cells of patients with SLE, which promotes the activation of CD4+ T cells and influences the differentiation of effector T cells, predominantly Treg cells. Moreover, METTL3 deficiency promotes antibody production and aggravates the lupus-like phenotype in mice. METTL3 enhances Foxp3 mRNA decay and reduces Foxp3 expression in an m^6^A-dependent manner, thereby inhibiting Treg cell differentiation. In summary, METTL3 triggers SLE pathogenesis by over-activating CD4+ T cells and dysregulating effector T-cell differentiation via stabilizing Foxp3 mRNA, which could serve as a potential target for therapeutic intervention in SLE. However, Liu et al. (75) reported that METTL3 expressions are elevated in patients with SLE. The upregulated METTL3 promotes IRF4 expression in an m^6^A-dependent manner, thus causing plasma cell infiltration-mediated kidney damage of SLE (**Table 3**).

#### 3.1.3 METTL3 and RA

RA is a systematic autoimmune disease characterized by chronic synovitis and joint destruction (76). Abnormal proliferation of synovial fibroblast (SF), also termed fibroblast-like synoviocyte (FLS), in the joints and the formation of granulations that erode and destroy the articular cartilage contributes to RA occurrence (77). The typical pathology of RA is the persistence of inflammation maintained by SF and is driven by multiple epi-modifications (78). In addition, fibroblasts can switch from early immunosuppressive to stimulatory in response to relevant factors to promote RA development (79).

SF are effector cells of synovial hyperplasia of RA and are regarded as tumor-like cells with characteristics such as abnormal proliferation, migration and invasion, resistance to apoptosis, and so on (80). SF plays an important role in the development and progression of RA. While promoting apoptosis or inhibiting proliferation, migration, and invasion of SF efficiently prevents RA occurrence and development. Single-cell analysis and machine learning found that the expression levels and enrichment pathways of METTL3 and insulin-like growth factor binding protein 2 (IGFBP2) are different among SF subgroups (64). Further investigation of receptor-ligand interactions found that NPR3 and GHR may have a role in SF interactions (64). As predicted by machine learning, IGFBP2, and METTL3 are identified as key factors regulating m^6^A on NPR3 and GHR (64). In summary, NPR3 and GHR, as well as IGFBP2 and METTL3 perform important regulatory functions in RA. This study provides a basis for future studies to identify pathogenic cell subpopulations and molecular mechanisms in RA. As a chronic inflammatory disorder, FA shows synovial hypertrophy, severe joint damage, and loss of joint function (76). The hyper-proliferating FLS releases a large number of pro-inflammatory indicators, such as IL-6, IL-1b, and TNF-a, causing abnormal inflammation in the synovial membrane (81). Moreover, the activated FLS release matrix metalloproteinases (MMPs), promote FLS migration and invasion (82). Shi et al. (65) and Su et al. (83) found that METTL3 expression is significantly upregulated in human RA synovial tissues and RA rat models. Inhibition of METTL3 suppresses IL-6, MMP-3, and MMP-9 levels in human RA-FLS and rat RA model-FLS (65). In contrast, METTL3 overexpression reverses these effects. Additionally, in FLS, METTL3 regulates the inflammatory response of FLS by activating the NF-kB signaling pathway (65). These findings collectively suggest that METTL3 triggers FLS activation and inflammatory response by targeting the NF-kB signaling pathway. Similarly, METTL3-mediated methylation of RAC2 contributes to cell motility, oxidative stress, and inflammation in TNF-α-stimulated RA fibroblast-like synovial cells (84). Therefore, targeting METTL3 in FLS is a promising therapeutic strategy for RA. As reported, a high level of TTC4 is associated with poor outcomes in patients with RA, which promotes the production of inflammatory factors and reduces the generation of anti-oxidant factors in the articular tissue of mice with RA (66). Lu et al. (66) demonstrated that inhibition of METTL3 reduces TTC4 mRNA stability and expression in articular tissue of mice with RA, which reduces inflammation and oxidative stress by promoting HSP70 expression that further targets NLRP3. In summary, targeted regulation of METTL3 reduces oxidative response and inflammation in the RA model by regulating the TTC4/HSP70/NLRP3 pathway. Therefore, targeting METTL3 is therapeutic for RA (**Table 3**).

Furthermore, seeking new drugs that suppress the pathological functions of RA-FLS has become a possible method for RA therapy. *Artemisinin* (ATT) is a well-known antimalarial drug extracted from the plant Artemisia annua L. with immunomodulatory effects, anti-viral effects, anticancer activities, and so on (85). The ATT derivatives of the natural compound ATT show broad anticancer activities without toxicity on normal tissues (86, 87). Recently, ATT has been reported to have the potential to treat RA. Chen et al. (67) reported that ATT administration restrains RA-FLS migration and invasion via suppressing epithelial-to-mesenchymal transition (EMT), as well as inhibiting RA-FLS proliferation and inducing RA-FLS apoptosis. Moreover, ATT treatment relieves RA in mice. RNA-sequencing analysis and bioinformatics analysis identify intercellular adhesion molecule 2 (ICAM2) as a promoter of RA progression in RA-FLS (67). ATT inhibits RA progression by suppressing ICAM2/PI3K/AKT/p300 pathway in RA-FLS. Moreover, ATT inhibits METTL3-mediated m^6^A methylation on ICAM2 mRNA in RA-FLS (67). Interestingly, p300 directly facilitates METTL3 transcription, which could be restrained by ATT in RA-FLS. Importantly, the expression of METTL3, ICAM2, and p300 in synovium tissues of patients with RA is related to clinical characteristics and therapy response (67). This study provides strong evidence that ATT has therapeutic potential for RA management by suppressing proliferation, migration, and invasion, in addition to inducing RA-FLS apoptosis through modulating the feedback loop of METTL3/ICAM2/PI3K/AKT/p300, supplying the fundamental basis for the clinical application of ATT in RA therapy. Moreover, METTL3, ICAM2, and p300 might serve as biomarkers for the therapy response of patients with RA (**Table 3**).

#### 3.1.4 METTL3 and pSS

Primary pSS is a chronic autoimmune disorder defined by xerostomia and keratoconjunctivitis sicca, genetic and epigenetic factors have been considered to affect pSS development (56). Glandular inflammation and tissue impairment eventually give rise to disturbances of secretory and clinical manifestations of dryness, including dry eye and xerostomia (88). pSS is a chronic inflammatory autoimmune disease and is characterized by exocrine gland impairment, such as the salivary and lacrimal glands, which could result in dry mouth and eye (89). Dysregulation of m^6^A modification is closely associated with pSS.

Peripheral blood mononuclear cells (PBMCs) are the effector cells of autoimmune disorders (90). Ma et al. (68) found that the expression level of m^6^A is markedly increased in the PBMCs of patients with pSS and dry eye. The relative mRNA and protein levels of METTL3 and YTHDF1 are markedly elevated in pSS patients with dry eye. The m^6^A RNA level is found to be positively related to METTL3 expression in patients with pSS. The m^6^A RNA level is associated with C4, while METTL3 mRNA expression is associated with C3. This work reveals that the upregulation of m^6^A and METTL3 is associated with the performance of serological indicators and dry eye signs in patients with pSS. METTL3 may contribute to the pathogenesis of pSS-related dry eye (**Table 3**).

#### 3.1.5 METTL3 and autoimmune uveitis

Autoimmune uveitis, a chronic and unpredictable autoimmune disease, is characterized by inflammatory cell infiltration affecting the retina and uvea, which leads to visual disability and even blindness (91). Study has demonstrated that autoreactive Th17 cells are the main drivers of autoimmune uveitis (92). Th17 cells can be categorized into two functionally distinct subsets: non-pathogenic and pathogenic Th17 cells (93). Th17 cells, generated in the presence of TGF-beta, IL-6, IL-17, and IL-10, are largely nonpathogenic (94). In contrast, IL-23 alone or together with IL-6 induces highly pathogenic Th17 cells, which express signature genes including IL-17A, IL-17F, IL-23 receptor (IL-23R), IL-1R1, and granulocyte-macrophage colony-stimulating factor (93). METTL3 maintains the homeostasis of Th17 cells. Zhao et al. (69) found that the expression of METTL3 is decreased, along with reduced m^6^A levels, in eyeballs and T cells of experimental autoimmune uveitis. Overexpression of METTL3 ameliorates the development of experimental autoimmune uveitis and suppresses pathogenic Th17 cell responses *in vivo* and *in vitro*. Mechanistically, METTL3 promotes the expression of ASH1L via enhancing its stability in an YTHDC2-dependent manner, which further decreases the expression of IL-17 and IL-23R, resulting in reduced pathogenic Th17 responses. Together, these data reveal a pivotal role of METTL3 in regulating pathogenic Th17 responses, which may contribute to human uveitis therapy (**Table 3**).

Pathogenic Th17 cells are critical drivers of autoimmune uveitis (95). Wei et al. (70) showed that miR-338-3p endows dendritic cells (DCs) with an increased ability to activate IRBP 1-20-specific Th17 cells by promoting the production of IL-6, IL-1beta, and IL-23. *In vivo*, administration of miR-338-infected DCs promotes pathogenic Th17 responses and exacerbates uveitis development. Dual-specificity phosphatase 16 (Dusp16) is a target of miR-338-3p (70). miR-338-3p represses Dusp16 and thereby strengthening the p38 signaling, resulting in increased production of Th17-polarizing cytokines and subsequent pathogenic Th17 responses (70). In addition, METTL3 mediates miR-338-3p upregulation in activated DCs. Together, these findings identify a vital role for METTL3/miR-338-3p/Dusp16/p38 signaling in DC-driven pathogenic Th17 responses and suggest a potential therapeutic avenue for uveitis and other Th17 cell-related autoimmune disorders (**Table 3**).

#### 3.1.6 METTL3 and MG

MG is an autoantibodies-mediated autoimmune disease with complications of deregulated levels of immune factors that are associated with anti-inflammation and pro-inflammation (58). Previous study indicates that the aberrant regulation of immune T cells and inflammatory factors are linked to MG occurrence (58). The NLRP3 inflammasome is present primarily in immune and inflammatory cells following activation by inflammatory stimuli (71). These cells include macrophages, monocytes, DCs, and splenic neutrophils. METTL3 has been reported to regulate the normal function of these immune cells, as well as involved in the regulation of NLRP3 inflammasome. Consistently, Li et al. (72) found that the serum expression level of PTX3 in patients with MG is up-regulated compared to the normal group. METTL3-mediated m^6^A modification decreases PTX3 stability to enhance its expression level, which subsequently induces the STAT3/NLRP3 inflammasome and promotes STAT3 synthesis, promoting inflammation and pyroptosis in patients with MG, as well as in MG mouse model. This study suggests that PTX3 promotes inflammation and pyroptosis by regulating the STAT3/NLRP3 inflammasome signaling pathway, which requires the modification of METTL3. Therefore, targeting METTL3 might be therapeutic for PTX3-induced MG (**Table 3**).

Collectively, these findings suggest that METTL3 is involved in the pathogenesis of autoimmune diseases. Therefore, we propose that METTL3 may be a valuable diagnostic biomarker and a promising therapeutic target for autoimmune diseases. Nonetheless, additional fundamental and clinical studies are warranted to consolidate the evidence supporting their association and clinical potential.

### 3.2 METTL3 and myelocytic leukemia

Some types of cancer directly result from the uncontrolled proliferation of immune cells. For instance, leukemia is caused by white blood cells, which are immune cells (96). Whereas lymphoma arises from lymphocytes, which are adaptive B or T cells (97). Abnormal expression of METTL3 has been extensively implicated in leukemia, including chronic myelocytic leukemia (CML) and acute myeloid leukemia (AML), where it functions as an oncogene or regulates other oncogenes.

#### 3.2.1 METTL3 and CML

**Figure 2:**
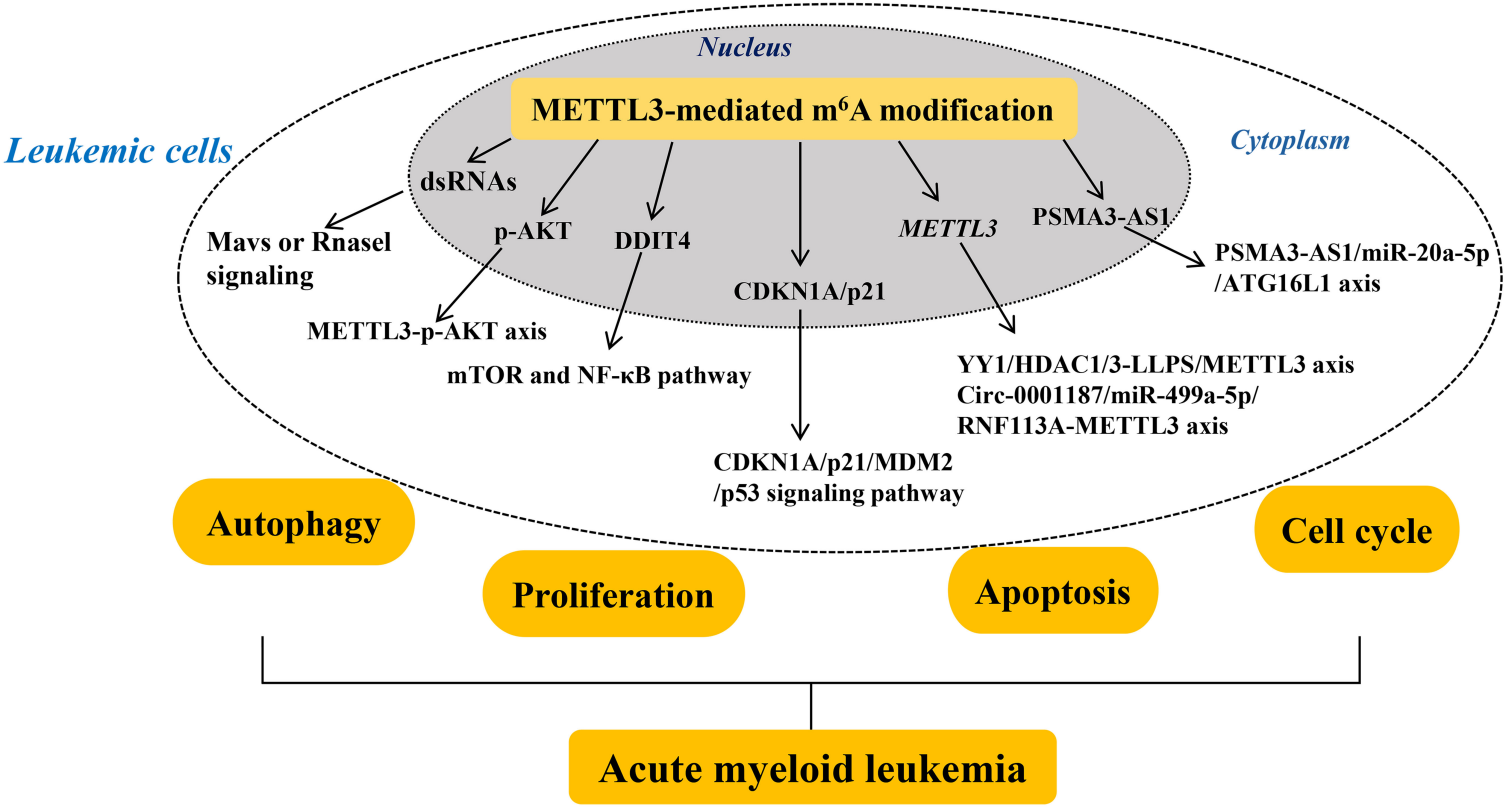
Mechanisms of METTL3’s role in myeloid leukemia. The roles and mechanisms of METTL3 in chronic and acute myeloid leukemia are thoroughly summarized. In summary, METTL3 contributes to the development of myeloid leukemia with different mechanism. Methyltransferase-like 3 (METTL3). Double-stranded RNAs (dsRNAs). Protein kinase B (AKT). Damage-inducible transcript 4 (DDIT4). Cyclin-dependent kinase inhibitor 1A (CDKN1A). Mouse double minute 2 (MDM2). Yin Yang 1 (YY1). YTH N^6^-methyladenosine RNA binding protein 1 (YTHDF1).

Abnormally upregulated METTL3 promotes oncogene pescadillo homolog 1 (PES1) translation directly, which enhances the proliferation, sensitivity to imatinib (a tyrosine kinase inhibitor), and resistance of CML cells (98). METTL3, acting as an oncoprotein in CML, specifically modifies PES1 transcripts through its cytoplasmic localization ability rather than its catalytic activity (98). This finding suggests that targeting METTL3 may be a promising therapeutic strategy for tyrosine kinase inhibitor-resistant CML. Lai et al. (99) demonstrated that METTL3 functions indirectly as a downstream target of LINC00470 to modify the oncogene PTEN, thereby suppressing CML cell autophagy and resulting in chemo-resistance. Besides, Yao et al. (100) demonstrated that METTL3-mediated m^6^A modification on the long non-coding RNA (lncRNA) nuclear-enriched abundant transcript 1 (NEAT1) effectively downregulates NEAT1 expression in CML, thereby promoting the proliferation and inhibiting the apoptosis of CML cells. Interestingly, the upregulation of miR-766-5p expression is observed in CML PBMCs, which potentially abolishes NEAT1 functions in CML cells (100). miR-766-5p exerts its function by inhibiting the expression of cyclin-dependent kinase inhibitor 1A (CDKN1A) in CML PBMCs. These findings suggest that in CML, METTL3 acts as an oncoprotein by directly targeting the NEAT1/miR-766-5p/CDKN1A axis to promote CML progression (**Figure 2**).

In summary, the overexpressed level of METTL3 is associated with CML by its direct or indirect oncogenic functions, suggesting that METTL3 is a promising biomarker for CML diagnosis or prognosis, as well as a potential therapeutic target for CML treatment.

#### 3.2.2 METTL3 and AML

AML is a hematologic malignancy characterized by dysregulated proliferation and accumulation of leukemic cells in the hematopoietic system (101). Gao et al. (102) reported that the deletion of METTL3 conditionally in murine fetal liver brings about hematopoietic failure and perinatal lethality. Importantly, loss of METTL3 induces the formation of endogenous dsRNAs that are characterized by high m^6^A modification in their native state, which further activates the aberrant innate immune response and results in hematopoietic defects by triggering the Mavs or Rnasel signaling (102). Furthermore, inhibition of Mavs or Rnasel signaling prevents the aberrant immune response and partially rescues the hematopoietic defects observed in METTL3-deficient cells *in vitro* and *in vivo*. These results suggest that METTL3-mediated m^6^A modification regulates the innate immune response during mammalian hematopoietic development (**Figure 2**).

Additionally, the abnormal differentiation of hematopoietic stem/progenitor cells (HSPCs) is a common feature of AML, which relates to the aberrantly low level of METTL3 (103). Loss of METTL3 enhances the p-AKT level, which contributes to the abnormal differentiation of HSPCs. Overall, METTL3 contributes to HSPC-induced AML by regulating p-AKT, providing a rationale for the therapeutic targeting of METTL3 in AML. As reported, Sun et al. (104) found that the downregulated level of METTL3 represents a novel prognostic factor and a promising therapeutic target for patients with ETV6/RUNX1-positive AML. Targeted suppression of METTL3 in the myeloid lineage reportedly confers protection against age-related and diet-induced non-alcoholic fatty liver disease (NAFLD) and obesity in mice and is accompanied by improved inflammatory and metabolic phenotypes (105). Loss of METTL3 leads to a marked elevation in the mRNA level of DNA damage-inducible transcript 4 (DDIT4) and a strong suppression of mTOR and NF-κB pathway in macrophages in response to cellular stress and cytokine stimulation (105). This indicates that METTL3-mediated m^6^A modification on DDIT4 mRNA results in macrophage metabolic reprogramming during NAFLD and obesity. These findings suggest that METTL3 is actively involved in leukemia (**Figure 2**).

Furthermore, the abnormally overexpressed METTL3 plays a critical role in AML progression, which relates to different development stages of AML. For example, Sang et al. (106) demonstrated that METTL3 expression is significantly upregulated in most patients with AML. Moreover, METTL3 overexpression is associated with shorter survival time in patients with AML because of its ability to promote cell proliferation and cell cycle progression. Conversely, suppression of METTL3 results in the upregulation of CDKN1A/p21 and reduces mouse double minute 2 (MDM2) mRNA stability, thereby activating the tumor protein 53 (p53) signaling pathway to promote cell proliferation while also facilitating apoptosis and differentiation. These findings suggest that elevated levels of METTL3 play an oncogenic role by targeting the CDKN1A/p21/MDM2/p53 signaling pathway in AML. Similarly, Li et al. (107) uncovered that Yin Yang 1 (YY1) is a transcription factor of METTL3 that interacts with the HDAC1/3 promoter region of METTL3 to promote METTL3 expression in a moderate liquid-liquid phase separation manner, which further enhances the proliferation of AML cells. Therefore, METTL3 acts as the oncogene for AML progression by regulating AML cell proliferation, which might be a biomarker for the developed AML diagnosis. Besides, Wu et al. (108) indicated that METTL3-mediated m^6^A modification on PSMA3-AS1 enhances the stability and level of PSMA3-AS1 in patients with FLT3-ITD+ AML, which in turn promotes AML cell autophagy by targeting miR-20a-5p that further downregulates ATG16L1 mRNA expression, ultimately promotes AML progression. Therefore, METTL3 enhances AML progression by promoting AML cell autophagy via the PSMA3-AS1/miR-20a-5p/ATG16L1 axis, which provides new horizons for early screening and targeted therapy of FLT3-ITD+ AML by targeting METTL3. Furthermore, circular RNAs (circRNAs), a class of non-coding RNAs, serve as important epigenetic regulators in AML progression (109). Large-scale sample detection found that circ-0001187 is only significantly downregulated in newly diagnosed patients with AML whereas is increased in patients with hematological complete remission compared with controls (110). Circ-0001187 sponges miR-499a- 5p to enhance the expression of E3 ubiquitin ligase RNF113A, which further mediates METTL3 ubiquitin/proteasome-dependent degradation to reduce METTL3 expression via K48-linked polyubiquitin chains. The inhibited level of METTL3 promotes proliferation and inhibits apoptosis of AML cells *in vitro* and *in vivo*. Therefore, METTL3 serves as the oncogenic target in AML, which is regulated by the circ-0001187/miR-499a-5p/RNF113A axis (**Figure 2**).

In summary, METTL3 contributes to the development of AML, suggesting its potential for AML diagnosis and classification.

#### 3.2.3 METTL3 and AML treatment failure

Although great advances have been made in AML therapy, treatment failure occurs in many patients with poor outcomes. Therefore, further investigation of the underlying mechanism for AML treatment failure is required, which might be helpful in exploring new methods to improve AML treatment outcomes.

AML is a distinct hematologic malignancy that targets a specific subpopulation of blood-forming stem/progenitor cells (111). Specifically, AML is characterized by the malignant proliferation of immature bone marrow stem cells in the bone marrow and peripheral blood (112). The adipogenic differentiation of bone marrow mesenchymal stem cells (BMSCs) confers chemoresistance to AML cells (111, 113). METTL3 plays a vital role in the sensitivity of AML cells to Cytarabine through its interaction with immune-infiltrating cells and autophagy (114). Besides, Pan et al. (115) observed a conspicuous reduction in METTL3 expression in BMSCs obtained from patients with AML, which stimulates BMSC adipogenesis and contributes to AML pathogenesis. The reduced level of METTL3 in BMSCs functions by mediating m^6^A modification on AKT1 transcripts to increase its mRNA stability and induce AKT1 protein expression, thereby activating the PI3K/AKT pathway that causes BMSC adipogenesis and chemoresistance in AML cells. These findings suggest that the METTL3-mediated m^6^A modification on AKT1 is responsible for AML chemoresistance induced by BMSC adipogenesis, hinting at the possibility of targeting METTL3 for BMSC adipogenesis regulation and AML chemoresistance removal. The homing/engraftment in bone marrow (BM) is a crucial step for AML cells to acquire chemoresistance by interacting with stromal cell components, which relates to the upregulated expression of METTL3 in chemoresistant cells (116). Specifically, METTL3-mediated m^6^A modification on ITGA4 mRNA induces ITGA4 expression, which further enhances the homing/engraftment of AML cell chemoresistance *in vitro* and *in vivo*. Importantly, inhibition of METTL3 improves homing/engraftment chemoresistance in AML cells. These results suggest that METTL3-mediated m^6^A modification functions in the interaction between AML cells and BM niches and clarify the relationship between METTL3 and AML homing/engraftment, suggesting a therapeutic strategy for the treatment of refractory/relapsed AML with METTL3 inhibitors (**Figure 2**).

*Adriamycin* chemoresistance poses a great threat in AML therapy (117). Transcriptome-wide analysis of RNA m^6^A modification in *Adriamycin*-resistant AML cells found that the expression level of METTL3 is elevated, which modifies m^6^A on genes that are enriched in pathways of the FoxO signaling pathway, p53 signaling pathway, and Notch signaling pathway (118). After a combined treatment of STM2457 (an inhibitor of METTL3) and *Adriamycin*, the proliferation of AML cells is inhibited. Thus, the abnormality of METTL3-mediated m^6^A modification plays an important role in AML *Adriamycin* resistance. Similarly, low levels of lncRNA maternally expressed gene 3 (MEG3) and miR-493-5p are associated with *Adriamycin* resistance and poor prognosis in patients with AML (119). Wang et al. (119) found that MEG3 leads to elevated expression of miR-493-5p that further targets METTL3. Subsequently, METTL3 elevates the m^6^A level of MYC to increase MYC expression, leading to *Adriamycin* resistance in AML cells. Importantly, suppression of METTL3 inhibits AML cell proliferation and promotes their apoptosis, improving *Adriamycin* sensitivity in AML cells. Collectively, MEG3 and miR-493-5p promote the *Adriamycin* resistance of AML cells by inactivating the METTL3/MYC axis (**Figure 2**).

In summary, METTL3 plays an oncogenic role in AML chemoresistance and is associated with poor outcomes in patients with AML. Therefore, we propose that targeting METTL3 represents a rational and promising therapeutic strategy for improving AML treatment failure.

### 3.3 METTL3 and skin cancers

The skin serves as a primary barrier against microbial invasion via the production and secretion of vital antimicrobial proteins by skin cells and the presence of immune cells in specific layers (120). It has been demonstrated that the monoclonal proliferation of T lymphocytes in the skin contributes to the onset of cutaneous T cell lymphomas (CTCL) (121). Wang et al. (122) reported that METTL3 is significantly downregulated in CTCL cells, which is responsible for CTCL cell proliferation and migration by promoting CDKN2A mRNA degradation in an m^6^A reader IGF2BP2 dependent way. This study suggests the participation of METTL3-mediated m^6^A modification in regulating the function of skin immune cells that trigger the onset and progression of skin cancers. However, the exact mechanism is still unclear and needs further exploration. Generally, uncontrolled cell proliferation and migration, cell cycle progression, as well as EMT, are indicative of aggressive processes for tumorigenesis and metastasis (123). Exploring the molecular mechanism for these processes from the perspective of METTL3-mediated m^6^A modification has been reported in skin cancers, which are highly aggressive malignancies originating in the skin and occur during the immunosuppression process, including melanoma, cutaneous squamous cell carcinoma (cSCC), acral melanoma, and keratinocyte transformation (124) (**Table 4**).

**Table 4:** Targets and functions of METTL3 in skin cancers.

METTL3			
Targets	Functions	Skin cancer	Reference
*CDKN2A mRNA*	Promotes *CDKN2A mRNA* degradation in an IGF2BP2 dependent way	**CTCL**	(122)
*METTL3mRNA*	Activates the PI3K/AKT pathway	**Melanoma**	(125)
*UCK2 mRNA*	Enhances *UCK2 mRNA* stability and expression		(126)
*MMP2 and* *N-cadherin mRNA*	Facilitates the accumulation of MMP2 and N-cadherin		(127)
*DNp63 mRNA*	Enhances *DNp63 mRNA* stability and expression	**cSCC**	(128)
*TXNDC5 mRNA*	Methylate on *TXNDC5 mRNA* directly	**Acral melanoma**	(129)
*PRDM2 mRNA*	Facilitates *PRDM2 mRNA* degradation	**Keratinocyte transformation**	(130)
*YY1 and MDM2 mRNA*	Augments the translation of *YY1* and *MDM2 mRNA*		(130)

A detailed summary and analysis of the targets and functions of METTL3 reveals its significant involvement in the processes of skin cancers. Specifically, METTL3 contributes to the overall development of skin malignancies by regulating targets differently.

Methyltransferase-like 3 (METTL3). Insulin-like growth factor binding protein 2 (IGFBP2). Cyclin-dependent kinase inhibitor 2A (CDKN2A). Uridine cytidine kinase 2 (UCK2). Matrix metalloproteinases (MMPs). Thioredoxin domain-containing protein 5 (TXNDC5). PR/SET domain 2 (PRDM2). Mouse double minute 2 (MDM2). Yin Yang 1 (YY1). YTH N^6^-methyladenosine RNA binding protein 1 (YTHDF1).

The bold words indicates the specific skin cancers.

METTL3 functions in these skin cancers either directly as the oncogene or indirectly by mediating RNA m^6^A modification on RNAs to regulate their fate and functions, including mRNA stability, nuclear processing, transport, localization, translation, primary microRNA processing, and RNA-protein interactions. Chang et al. (125) demonstrated that METTL3 is a direct target gene of miR-302a-3p in melanoma cells, which upregulates METTL3 expression. The extensive expression of METTL3 further activates the PI3K/AKT pathway in melanoma cells, which subsequently facilitates cell proliferation, invasion, and cell cycle progression, as well as EMT marker expression, while suppressing apoptosis. These findings suggest that METTL3 functions directly as the oncogene to facilitate melanoma metastasis by regulating the METTL3-PI3K/AKT axis. Moreover, uridine cytidine kinase 2 (UCK2) is an oncogene that is associated with poor survival in patients with melanoma. Wu et al. (126) reported that the extensively increased expression of METTL3 enhances UCK2 mRNA stability and expression by mediating m^6^A modification on UCK2 transcripts; this subsequently promotes melanoma progression by regulating the WNT/beta-catenin pathway. Therefore, METTL3 functions indirectly by targeting oncogene UCK2, which is a promising target for preventing and treating melanoma. In addition, the MMP family contributes to the metastasis of melanoma cells. Dahal et al. (127) reported that the elevated levels of METTL3 in melanoma cells typically facilitate the accumulation of MMP family members such as MMP2 and N-cadherin. Remarkably, the upregulation of METTL3 with a catalytic site mutation result in increased expression of MMP2 and N-cadherin, thereby indicating that the METTL3-mediated m^6^A modification of MMP2 and N-cadherin contributes to colony formation and invasion of melanoma cells. These findings highlight that METTL3 plays an important role in human melanoma invasion by targeting oncogenes MMP2 and N-cadherin (**Table 4**).

Furthermore, Zhou et al. (128) found that METTL3-mediated RNA m^6^A modification plays an oncogenic role in cSCC, and the same is evidenced by the upregulation of both METTL3 and m^6^A, together with DNp63 expression. This highlights that METTL3 has a critical role in regulating cSCC tumorigenesis by targeting DNp63. Another study revealed that METTL3 expression is upregulated via direct methylation on thioredoxin domain-containing protein 5 (TXNDC5) mRNA in acral melanoma tissues and this upregulation is associated with advanced stages in patients with primary acral melanoma (129). This suggests that METTL3 promotes acral melanoma progression by targeting TXNDC5 and highlights the m^6^A-dependent METTL3 signaling pathway as a promising therapeutic strategy for treating patients under this condition. Additionally, Zhao et al. (130) demonstrated that an elevated level of METTL3 triggers the transformation of human keratinocytes through p53 pathway deactivation. METTL3 collaborates with YTHDF1 to facilitate the degradation of PR/SET domain 2 (PRDM2) mRNA (a positive regulator of p53) and with YTHDF1 to augment the translation of YY1 and MDM2 mRNA (a negative regulator of p53). Briefly, METTL3 facilitates p53 signaling in human keratinocytes by deactivating the YTHDF2/PRDM2 axis and activating the YTHDF1/YY1-MDM2A axis (**Table 4**).

### 3.4 METTL3 and anticancer immunotherapy

An emerging concept posits that cancer progression may be partially attributable to the escape of cancer cells from immune surveillance (131). Immune evasion may arise from an overabundance of inhibitory immune cells or cytokines and other poorly understood factors (132). A comprehensive pan-cancer analysis suggests that a high level of METTL3 in most cancers usually is indicative of poor outcomes and low disease-free survival rates (133). Importantly, METTL3 levels are strongly linked to immune cell infiltration and immune checkpoint gene levels, which regulate the tumor immune microenvironments and EMT via modulating RNA modification and metabolism (133). Specifically, METTL3 plays a vital role in the sensitivity of AML cells to Cytarabine through its interaction with immune-infiltrating cells and autophagy (114). Therefore, METTL3 serves as an oncogene in most cancers and shows potential as a prognostic biomarker and a potential therapeutic target. As reported, the knockdown of METTL3 and METTL14 augments the efficacy of PD-L1 treatment in colorectal cancer and melanoma, boosting the infiltration of cytotoxic CD8+ T cells into tumor tissues and elevating the secretion of IFN-c, CXCL9, and CXCL10 within the tumor-associated macrophages (134). Mechanistically, knockdown of either METTL3 or METTL14 promotes IFN-C/IFN-C/STAT1/IRF1 signaling by stabilizing STAT1 and IRF1 mRNAs via an YTHDF2-dependent mechanism. Moreover, m^6^A-related lncRNA TRAF3IP2-AS1 predicts prognosis and designs immunotherapy in patients with AML (135). Therefore, METTL3-mediated RNA m^6^A modification plays a crucial role in adaptive immunity and is a novel mechanism underlying the failure of cancer immunotherapy. Targeting METTL3 may hold promise as a potential therapeutic strategy for improving anticancer immunotherapy.

## 4 Discussion

METTL3 and its role in RNA m^6^A modification are intricately linked to the development and progression of immune dysfunctions in humans (136). This review aims to provide a thorough overview, categorization, and analysis of research surrounding METTL3, elucidating its functions, underlying mechanisms, applications, and future prospects in immune disorders, which encompass antiviral immune suppression (due to viral infections) and disruptions in immune tolerance (related to autoimmune diseases, myeloid leukemia, skin cancers, and anticancer immunotherapy).

The findings suggest that METTL3-mediated RNA m^6^A modification plays a significant role in immune system irregularities. Elevated levels of METTL3 are associated with infections during periods of antiviral immune suppression, particularly enhancing the replication of enteroviruses such as PEDV, rotavirus, and EV71. In this context, METTL3 mainly influences inflammation and creates a supportive microenvironment, while its downregulation aids in the recovery of antiviral innate immunity and the clearance of viruses. Consequently, heightened METTL3 levels may act as both a diagnostic and prognostic biomarker and represent a promising target in the context of infections. Conversely, during immune tolerance breakdown, METTL3 can function as an oncogene, either directly or indirectly targeting other oncogenes, thereby driving the onset and advancement of autoimmune diseases, myelocytic leukemia, and skin cancers, as well as contributing to the ineffectiveness of anticancer immunotherapy. The mechanisms through which METTL3 operates in immune dysfunction include the modulation of inflammation, oxidative stress, autophagy, cell proliferation, apoptosis, and the cell cycle. Overall, this review underscores METTL3 as a significant factor in triggering immune dysfunctions.

However, research by Barbieri et al. (137) indicates that METTL3 is essential for the proliferation of AML cells, with its downregulation hindering the cell cycle and differentiation of these cells. This effect is mediated through its association with chromatin and its localization to the transcription start site of active genes that bind the CAATT-box binding protein CEBPZ. Collectively, these contrasting observations suggest that METTL3 regulates a novel chromatin-based pathway vital for sustaining the AML state and highlights it as a promising therapeutic target for preventing AML progression. Additionally, the findings concerning METTL3’s expression and function in the pathogenesis of CML and AML exhibit inconsistencies, likely due to differences in sample selection. Notwithstanding these disparities, the majority of studies support the oncogenic role of METTL3 in the progression of myelocytic leukemia. In terms of its implications in abnormal immune conditions, METTL3 holds potential as both a diagnostic and prognostic biomarker for distinguishing between CML and AML, as well as representing a promising therapeutic target for myelocytic leukemia. Nonetheless, further extensive data from both basic and clinical research are necessary to strengthen the evidence supporting their association and clinical significance.

## 5 Future research direction

Exploring the dual roles of METTL3 in both diagnostic and therapeutic contexts opens a wealth of research opportunities. One significant avenue for future research is the development of METTL3-targeting therapies. Given its implication in various cancers and autoimmune diseases, targeted inhibition or modulation of METTL3 activity could provide a novel therapeutic strategy. For instance, small molecule inhibitors or RNA-based therapies could be designed to specifically target METTL3, potentially halting the progression of diseases where METTL3 acts as an oncogene (138–141). Moreover, the precise mechanisms by which METTL3 contributes to the breakdown of immune tolerance and subsequent disease progression warrant further investigation. Understanding these pathways could reveal additional therapeutic targets and refine existing treatments. For example, identifying co-factors or downstream effectors of METTL3 could help in developing combination therapies that enhance the efficacy of current treatments or mitigate side effects. Another critical research direction involves the validation of METTL3 as a diagnostic and prognostic biomarker. Large-scale clinical trials are essential to establish the reliability and specificity of METTL3 levels in predicting disease onset, progression, and patient outcomes. Such trials should encompass a diverse patient population to ensure the generalizability of the findings. Furthermore, incorporating advanced techniques such as single-cell RNA sequencing and CRISPR-based gene editing could provide deeper insights into the role of METTL3 at the cellular and molecular levels. Beyond oncology and autoimmune diseases, the role of METTL3 in infectious diseases presents another promising research frontier. Investigating how METTL3 modulates the host immune response to various pathogens could uncover novel therapeutic targets and improve our understanding of host-pathogen interactions. For instance, exploring the interplay between METTL3 and viral replication mechanisms could lead to the development of antiviral therapies that exploit this relationship. In addition to these specific areas, there is a broader need to understand the context-dependent functions of METTL3. Its role as both an oncogene and a regulator of immune response highlights the complexity of its functions and necessitates a nuanced approach to therapeutic development. Research should aim to delineate the conditions under which METTL3 acts beneficially versus detrimentally, potentially leading to personalized medicine approaches where METTL3-targeting therapies are tailored to individual patients based on their specific disease context and METTL3 expression profiles. Furthermore, the potential off-target effects and long-term consequences of METTL3 inhibition need to be thoroughly evaluated. Preclinical studies in animal models and subsequent phase I/II clinical trials will be crucial for assessing the safety and efficacy of METTL3-targeting therapies. These studies should also explore the potential for resistance development and identify biomarkers that can predict patient response to treatment. In conclusion, METTL3 represents a multifaceted target with significant implications for both diagnostics and therapeutics. Expanding our understanding of its roles across different diseases and contexts will be key to unlocking its full potential. Future research should focus on elucidating the detailed mechanisms of METTL3 action, validating it as a biomarker in clinical settings, and developing targeted therapies that can be integrated into personalized treatment regimens. Through these efforts, we can better harness the power of METTL3 to improve patient outcomes across a range of diseases.

## 6 Conclusion

In summary, this review offers an in-depth interpretation of the connection between METTL3 and immune-related pathological events, while also underscoring its potential clinical applications. Therefore, it sheds light on existing gaps in the literature that need to be addressed, serving as a valuable reference for both researchers and clinicians.
