# Supplementary figures and images for: Methyltransferase-like 3-mediated RNA N6-methyladenosine contributes to immune dysregulation: diagnostic biomarker and therapeutic target
Source: Front Immunol. 2025 Mar 24;16:1523503. doi: 10.3389/fimmu.2025.1523503 (PMC11973086; doi:10.3389/fimmu.2025.1523503)

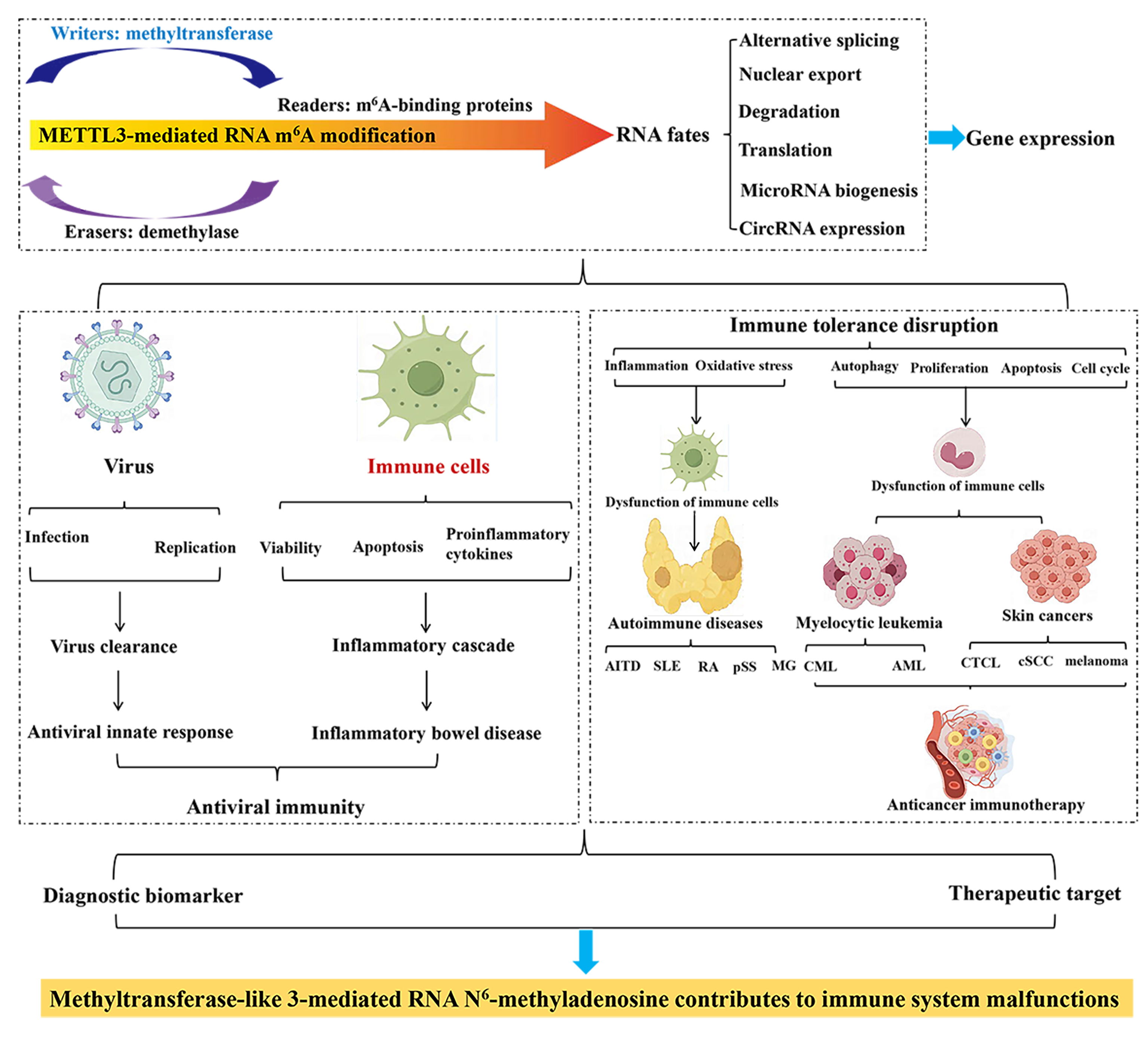

Supplement: Supplementary file 1 [file Image1.jpeg]
